# Differential gene expression of human chondrocytes cultured under short-term altered gravity conditions during parabolic flight maneuvers

**DOI:** 10.1186/s12964-015-0095-9

**Published:** 2015-03-20

**Authors:** Markus Wehland, Ganna Aleshcheva, Herbert Schulz, Katrin Saar, Norbert Hübner, Ruth Hemmersbach, Markus Braun, Xiao Ma, Timo Frett, Elisabeth Warnke, Stefan Riwaldt, Jessica Pietsch, Thomas Juhl Corydon, Manfred Infanger, Daniela Grimm

**Affiliations:** Clinic for Plastic, Aesthetic and Hand Surgery, Otto-von-Guericke University, 39120 Magdeburg, Germany; Max-Delbrück-Center for Molecular Medicine, 13092 Berlin, Germany; University of Cologne, Cologne Center for Genomics (CCG), 50931 Cologne, Germany; DLR German Aerospace Center, Biomedical Research, Gravitational Biology, 51147 Köln, Germany; Institute for Molecular Physiology and Biotechnology of Plants (IMBIO), University of Bonn, Gravitational Biology Group, 53115 Bonn, Germany; Department of Biomedicine, Aarhus University, Wilhelm Meyers Allé 4, DK-8000 Aarhus C, Denmark; German Aerospace Center (DLR), Institute of Aerospace Medicine, Biomedical Research, 51147 Köln, Germany

**Keywords:** Chondrocytes, Gene expression, Microgravity, Hypergravity, Vibration, Cytokines

## Abstract

**Background:**

Chondrocytes are the main cellular component of articular cartilage. In healthy tissue, they are embedded in a strong but elastic extracelluar matrix providing resistance against mechanical forces and friction for the joints. Osteoarthritic cartilage, however, disrupted by heavy strain, has only very limited potential to heal. One future possibility to replace damaged cartilage might be the scaffold-free growth of chondrocytes in microgravity to form 3D aggregates.

**Results:**

To prepare for this, we have conducted experiments during the 20th DLR parabolic flight campaign, where we fixed the cells after the first (1P) and the 31st parabola (31P). Furthermore, we subjected chondrocytes to isolated vibration and hypergravity conditions. Microarray and quantitative real time PCR analyses revealed that hypergravity regulated genes connected to cartilage integrity (*BMP4, MMP3, MMP10, EDN1, WNT5A, BIRC3*). Vibration was clearly detrimental to cartilage (upregulated inflammatory *IL6* and *IL8*, downregulated growth factors *EGF, VEGF, FGF17*). The viability of the cells was not affected by the parabolic flight, but showed a significantly increased expression of anti-apoptotic genes after 31 parabolas. The IL-6 release of chondrocytes cultured under conditions of vibration was not changed, but hypergravity (1.8 *g*) induced a clear elevation of IL-6 protein in the supernatant compared with corresponding control samples.

**Conclusion:**

Taken together, this study provided new insights into the growth behavior of chondrocytes under short-term microgravity.

**Electronic supplementary material:**

The online version of this article (doi:10.1186/s12964-015-0095-9) contains supplementary material, which is available to authorized users.

## Background

Joint friction at the extremities of long bones is reduced by articular cartilage. This kind of tissue is highly specialized, avascular, not innervated and consists mainly of a single cell type: the chondrocytes. The chondrocytes are tightly embedded in an extracellular matrix (ECM), which is composed of a network of collagens (predominantly collagen type II) and aggrecan. The collagen network contributes to the strength and mechanical resistance of cartilage tissue, whereas the aggrecan, a proteoglycan, comprising such molecules as chondroitin sulfate or keratin sulfate, is responsible for its viscoelasticity and flexibility due to its ability to absorb and retain considerable amounts of water [1-3]. Degenerative diseases of the cartilage like osteoarthritis are characterized by a progressive degradation of the ECM, caused by the increased secretion of matrix metalloproteinases (MMP) [4,5]. This process is triggered by pro-inflammatory cytokines, such as tumor necrosis factor-α (TNF-α) or interleukin-1β (IL-1β), which are transported into the cartilage via the synovial fluid [6,7]. The absence of vasculature and the extremely limited influx of chondrocyte progenitor cells [8] limit the tissues healing and restorative potential, so that in advanced stages of osteoarthritis a surgical replacement of the affected joint with a prosthesis is usually necessary.

Apart from the influences of age and use intensity, other factors have been found to mediate cartilage integrity. Most notably, microgravity (μ*g*) has a strong impact on cartilage. It has been reported, that astronauts, after staying a longer time in Space, suffer from a reduction of cartilage mass [9] due to mechanical unloading. In addition, when cultured in Space, cartilage tissue showed a reduced aggrecan density and a less-organized collagen subtype 2 organization hinting towards an impaired resistance to mechanical stress and breaking [10,11]. It is therefore of high interest, to study the impact of microgravity on chondrocytes. On the one hand, this will help to understand the detrimental effects of prolonged stays in Space on the cartilage, but on the other it might also help to find ways to counteract this phenomenon in Space as well as to ameliorate cartilage problems caused by wear on Earth in the future.

Microgravity (μ*g*) provides unique conditions for cell and tissue growth. It has been shown on various cell types, including chondrocytes, that cultivation under conditions of μ*g* can induce the formation of 3D aggregates. These aggregates are especially interesting, as they do not require any potentially interfering scaffolding like those being generated under normal gravity conditions [12-16].

Cell cultivation in Space, however, is an extremely complicated and expensive venture. Therefore, pre-studies such as a parabolic flight, providing 31×22 s of short-term real μ*g*, or simulated μ*g* on ground-based facilities such as the rotating wall vessel bioreactor, clinostats or the random positioning machine (RPM) have been established [17-19]. Especially the parabolic flight is an attractive method to achieve real μ*g* without going into Space. It should be taken into consideration, however, that every μ*g*-phase is flanked by two 20 s-lasting hypergravity phases of 1.8 *g*. Moreover, during the flight vibrations occur and have to be taken into account for interpretation of the results [20].

This study aimed to investigate the influence of short-term real μ*g* during a parabolic flight campaign on the gene expression profiles of cultivated chondrocytes. In addition, the effects of 1.8 *g* hypergravity as well as of vibration in an extent comparable to those during a parabolic flight were separately investigated.

## Results

### Influence of hypergravity on chondrocyte gene expression

Using the Microarray analysis technique, we found a total of 210 genes (top 30 are listed in Table 1, for a complete list see Additional file 1) which were significantly differentially expressed and showed a Fold-Change of >2 or < −2 under conditions of hypergravity of 1.8 *g*. An enrichment analysis for Gene Ontology (GO) Biological Processes (BP) terms (top 15 given in Table 2, for a complete list see Additional file 1) revealed that mainly development-related processes were detected. GO:0048598 (embryonic morphogenesis, p = 2.28×10^−5^), GO:0001501 (skeletal system development, p = 3.33×10^−5^), and GO:0048729 (tissue morphogenesis, p = 3.83×10^−5^) were the most prominent. However, many genes belonging to these BP are also implicated in cartilage development (GO:0051216, p = 5.59×10^−4^) or cell adhesion (GO:0007155, p = 5.96×10^−4^) and biological adhesion (GO:0022610, p = 6.05×10^−4^), such as the bone morphogenetic protein 4 (*BMP4*), fibroblast growth factor 9 (*FGF9*), hyaluronan and proteoglycan link protein 1 (*HAPLN1*), collagen type 2 α 1 (*COL2A1*), or laminin α 5 (*LAMA5*).

**Table 1:** **Top 30 most differentially expressed genes under hypergravity as detected by microarray analysis**

**Symbol**	**p-value**	**Fold-Change (1.8** ***g*** **vs. 1** ***g*** **)**
*ANGPTL4*	3,41336E-05	4,82382
*ADAM19*	1,31184E-03	3,65686
*LOC401233*	3,46212E-05	3,59384
*ARHGDIB*	6,38141E-06	3,37183
*TAGLN*	4,66727E-04	3,34324
*CTAG2*	4,76891E-05	3,28068
*PRG4*	2,57465E-04	3,27652
*CXCL12*	3,59100E-04	3,20598
*LAMC2*	4,52723E-05	3,09657
*CXCL12*	3,10216E-05	3,09447
*RAGE*	4,92007E-04	3,07122
*PRG4*	2,73879E-04	3,01963
*MSLN*	1,29948E-06	2,95963
*SRGN*	6,35801E-05	2,93607
*ZNF185*	7,53193E-06	2,92161
*MT1F*	5,01022E-06	-3,61603
*FGFBP2*	1,36152E-04	-3,68509
*COL2A1*	1,85922E-04	-3,70943
*HYAL1*	1,90804E-05	-3,83367
*MMP3*	7,88772E-05	-3,88035
*LRRC32*	1,77468E-05	-3,88223
*SMOC2*	2,93763E-05	-3,89646
*SERPINA3*	4,50468E-06	-4,56156
*STMN2*	4,95960E-04	-4,71568
*OGN*	2,69302E-05	-4,95328
*DLK1*	2,78088E-05	-5,78629
*GSTM1*	6,42006E-06	-7,46130
*FOXQ1*	5,44745E-05	-9,50597
*GSTM1*	3,27945E-07	-9,80354
*TMEM119*	1,61898E-06	-13,59590

**Table 2:** **Top 15 significantly enriched Gene Ontology biological processes under hypergravity**

**GO ID**	**Process**	**p-value**	**Genes represented**
GO:0048598	embryonic morphogenesis	2.28*10^−5^	*BMP4, WNT5A, FGF9, GDF5, EDN1, COL2A1, HOXD10, MSX1, LAMA5, ALDH1A3, TFAP2A, TXNRD1, FBN2*
GO:0001501	skeletal system development	3.33*10^−5^	*BMP4, WNT5A, AEBP1, FGF9, TUFT1, EDN1, COL2A1, DLK1, HOXD10, HOXC8, MSX1, TFAP2A, GPNMB*
GO:0048729	tissue morphogenesis	3.83*10^−5^	*BMP4, FOXQ1, CRYGS, PGF, LAMA5, ALDH1A3, TGM3, TFAP2A, TXNRD1, CA2*
GO:0048736	appendage development	4.16*10^−5^	*WNT5A, MSX1, MEOX2, FGF9, GDF5, COL2A1, FBN2, HOXD10*
GO:0060173	limb development	4.16*10^−5^	*WNT5A, MSX1, MEOX2, FGF9, GDF5, COL2A1, FBN2, HOXD10*
GO:0035295	tube development	1.79*10^−4^	*WNT5A, BMP4, FGF9, PGF, LAMA5, EDN1, HOPX, TFAP2A, HHIP, CXCL12*
GO:0035107	appendage morphogenesis	2.81*10^−4^	*WNT5A, MSX1, FGF9, GDF5, COL2A1, FBN2, HOXD10*
GO:0035108	limb morphogenesis	2.81*10^−4^	*WNT5A, MSX1, FGF9, GDF5, COL2A1, FBN2, HOXD10*
GO:0002009	morphogenesis of an epithelium	3.13*10^−4^	*BMP4, CRYGS, PGF, LAMA5, ALDH1A3, TFAP2A, CA2*
GO:0001525	angiogenesis	4.01*10^−4^	*BMP4, MEOX2, FGF9, PGF, LAMA5, EDN1, CXCL12, ANGPTL4*
GO:0060541	respiratory system development	4.49*10^−4^	*WNT5A, BMP4, FGF9, LAMA5, ALDH1A3, HOPX, HHIP*
GO:0051216	cartilage development	5.59*10^−4^	*WNT5A, BMP4, MSX1, FGF9, EDN1, COL2A1*
GO:0007155	cell adhesion	5.96*10^−4^	*HAPLN1, AEBP1, EPDR1, CPXM2, COL2A1, CDH2, EMILIN2, CXCL12, OMD, PGM5, LAMA5, ITGB1BP1, MSLN, LAMC2, GPNMB, THBS2, NTM*
GO:0022610	biological adhesion	6.05*10^−4^	*HAPLN1, AEBP1, EPDR1, CPXM2, COL2A1, CDH2, EMILIN2, CXCL12, OMD, PGM5, LAMA5, ITGB1BP1, MSLN, LAMC2, GPNMB, THBS2, NTM*
GO:0048514	blood vessel morphogenesis	6.81*10^−4^	*BMP4, MEOX2, FGF9, PGF, LAMA5, EDN1, CDH2, CXCL12, ANGPTL4*

### Influence of vibration on chondrocyte gene expression

**Figure 1:**
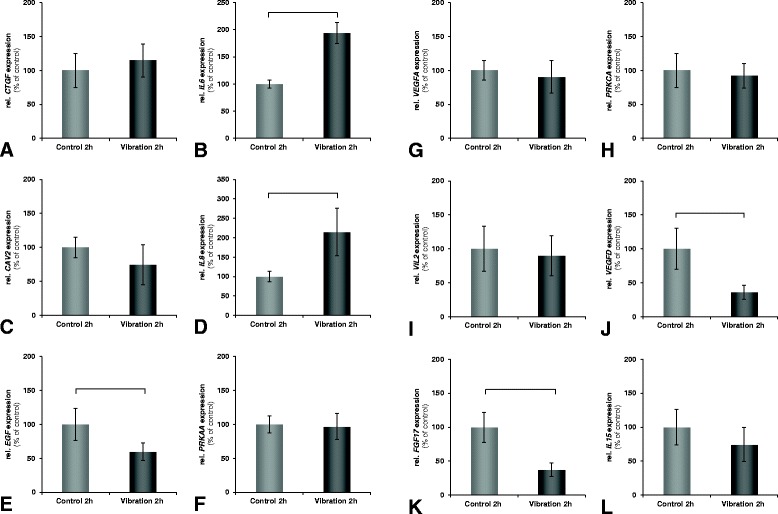
**Quantitative real-time PCR analysis of chondrocytes exposed to the Vibraplex device.** The Vibraplex device provides vibration profiles corresponding to those occurring during parabolic flight and allows the isolated analysis of their effects on cultivated cells. In all diagrams, the x-axis represents the experiment conditions and the y-axis the relative gene expression in % of control. Results are given as mean value ± SD. Significant changes (*p* < 0.05) are indicated by brackets above the bars. The analyzed genes were **A**: *CTGF*; **B**: *IL6*; **C**: *CAV2*; **D**: *IL8*; **E**: *EGF*; **F**: *PRKAA*; **G**: *VEGFA*; **H**: *PRKCA*; **I**: *VIL2*; **J**: *VEGFD*; **K**: *FGF17*; **L**: *IL15.*

Selected genes were analysed by quantitative real-time PCR. After 2 hours of vibration, gene expression of interleukin-6 (*IL6*, Figure 1B) and −8 (*IL8*, Figure 1D), were significantly elevated, while epidermal growth factor (*EGF*, Figure 1E), vascular endothelial growth factor D (*VEGFD*, Figure 1J), and fibroblast growth factor 17 (*FGF17*, Figure 1K) transcripts were significantly downregulated (p<0.05). No changes were observed for connective tissue growth factor (*CTGF*, Figure 1A), caveolin 2 (*CAV2*, Figure 1C), protein kinase, AMP-activated, α 1 (*PRKAA*, Figure 1F), vascular endothelial growth factor A (*VEGFA*, Figure 1G), protein kinase c alpha (*PRKCA*, Figure 1H), ezrin (*VIL2*, Figure 1I), and interleukin-15 (*IL15*, Figure 1L).

### Influence of hypergravity and vibration on soluble factor release

**Figure 2:**
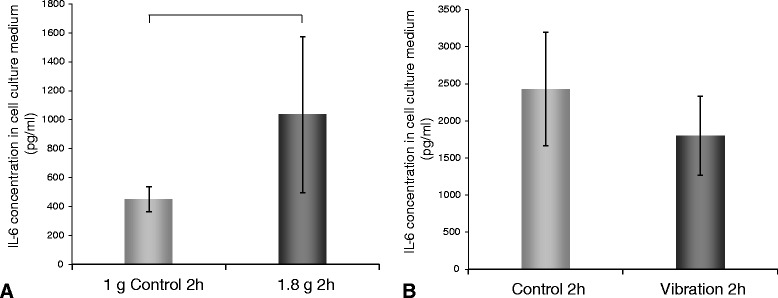
**ELISA analysis of IL-6 released in the cell culture supernatant. A**: IL-6 concentraion in the cell culture medium (pg/ml) from the hypergravity experiments. A significant increase in the secretion of IL-6 is detectable in cells cultured on the SAHC compared with static 1 *g* samples. **B**: IL-6 concentration in the cell culture medium (pg/ml) from the vibration experiments compared to the control sample. No significant changes could be detected. Significant changes (p<0.05) are indicated by brackets above the bars.

Hypergravity induced a more than 2-fold increase in the release of IL-6 in the supernatant (Figure 2A). IL-8, EGF, VEGFD and FGF17 concentrations in the culture supernatant of hypergravity samples were below the detection limit.

ELISA analysis revealved no significant change in the concentration of IL-6 protein in chondrocytes cultured under conditions of vibration (Figure 2B), whereas IL-8, EGF, VEGFD and FGF17 concentrations in the culture supernatant were below the detection limit.

### Parabolic flight maneuvers induced expression changes in chondrocytes

**Figure 3:**
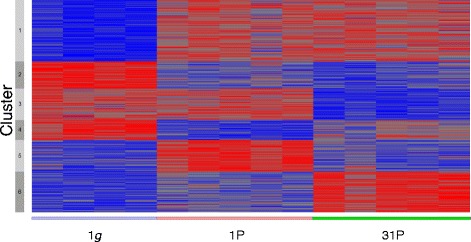
**Heatmap showing the clustering of differentially expressed transcripts for the parabolic flight microarray experiments.** Analysis after one (1P) and 31 parabolas (31P); red: strong expression; blue: weak expression.

The influence of the parabolic flight after 1P and 31 P was investigated. Shortly, the 1 *g* vs. first parabola (1P) vs. 31st Parabola (31P) set was subjected to an F-test. Resulting significant differential expressed probes (5% FDR) were clustered using k = 6. Individual expression characteristics of the 6 clusters are documented in Figure 3.

### Influence of the parabolic flight

#### Gene array analysis

Cluster 1 (Table 3) of the parabolic flight set consists of 112 genes, which were upregulated after both 1P and 31P. The predominant biological processes (Additional file 1, for all following clusters) which were found to be significantly enriched were mostly transcription- and metabolism-related (GO:0045449 regulation of transcription, p = 6.08×10^−8^; GO:0051252 regulation of RNA metabolic process, p = 1.59×10^−7^; GO:0006355 regulation of transcription, DNA-dependent, p = 1.48×10^−6^; GO:0006357 regulation of transcription from RNA polymerase II promoter, p = 8.69×10^−6^; GO:0006350 transcription, p = 1.09×10^−5^; GO:0019220 ~ regulation of phosphate metabolic process, p = 6.90×10^−5^). Interestingly, there was also anti-apoptosis (GO:0006916, p = 1.42×10^−4^), represented by such genes as vascular endothelial growth factor A (*VEGFA*), neurogenic locus notch homolog protein 2 (*NOTCH2*), or high-mobility group protein B1 (*HMGB1*).

**Table 3:** **Overview of the top 5 significantly enriched Gene Ontology biological processes in the 6 clusters of significantly differentially expressed genes during conditions of parabolic flight**

**Cluster**	**GO ID**	**Process**	**p-value**	**Genes represented**
**1**	GO:0045449	regulation of transcription	6.08*10^−8^	*HMGB1, SBNO2, ELF4, FOXO1, NFKB2, TRIB1, LIF, TSC22D1, HEXIM1, ZNF697, PER2, SIK1, SERTAD2, NFATC1, MAFG, BMP2, EGR2, CEBPB, KLF9, KLF13, RELB, LOC100131261, PURB, FOXN3, SOD2, PTHLH, NOTCH2, PHF1, ETS1, JMJD6, VEGFA, ERN1, FOXC2, HABP4, ZBTB2, NCOR2*
	GO:0051252	regulation of RNA metabolic process	1.59*10^−7^	*HMGB1, SBNO2, ELF4, FOXO1, MAPKAPK2, NFKB2, LIF, TSC22D1, HEXIM1, PER2, SIK1, NFATC1, SERTAD2, MAFG, BMP2, EGR2, CEBPB, KLF9, KLF13, RELB, FOXN3, PURB, SOD2, NOTCH2, JMJD6, ETS1, VEGFA, FOXC2, NCOR2*
	GO:0006355	regulation of transcription, DNA-dependent	1.48*10^−6^	*HMGB1, SBNO2, ELF4, FOXO1, NFKB2, LIF, TSC22D1, HEXIM1, PER2, SIK1, NFATC1, SERTAD2, MAFG, BMP2, EGR2, CEBPB, KLF9, KLF13, RELB, FOXN3, PURB, SOD2, NOTCH2, ETS1, VEGFA, FOXC2, NCOR2*
	GO:0006357	regulation of transcription from RNA polymerase II promoter	8.69*10^−6^	*HMGB1, BMP2, EGR2, CEBPB, KLF9, ELF4, KLF13, FOXO1, SOD2, LIF, ETS1, HEXIM1, VEGFA, FOXC2, SIK1, NCOR2*
	GO:0006350	transcription	1.09*10^−5^	*SBNO2, ELF4, FOXO1, NFKB2, TSC22D1, HEXIM1, ZNF697, PER2, NFATC1, SERTAD2, MAFG, EGR2, CEBPB, KLF9, KLF13, RELB, FOXN3, LOC100131261, PURB, NOTCH2, PHF1, JMJD6, ETS1, ERN1, HABP4, FOXC2, ZBTB2, NCOR2*
**2**	GO:0045449	regulation of transcription	6.34*10^−4^	*TXNIP, ZNF84, RCOR2, PPM1A, NFYA, ZNF514, ZNF512, MEN1, LHX2, ZNF239, ZNF471, ZNF599, THAP11, RNF14*
	GO:0006350	transcription	1.44*10^−3^	*TXNIP, ZNF84, RCOR2, LHX2, ZNF239, ZNF471, ZNF599, NFYA, ZNF514, THAP11, ZNF512, RNF14*
	GO:0007498	mesoderm development	9.48*10^−3^	*NUP133, OSR1, LHX2*
	GO:0006355	regulation of transcription, DNA-dependent	1.90*10^−2^	*MEN1, ZNF84, LHX2, PPM1A, ZNF471, ZNF599, NFYA, ZNF514, RNF14*
	GO:0051252	regulation of RNA metabolic process	2.16*10^−2^	*MEN1, ZNF84, LHX2, PPM1A, ZNF471, ZNF599, NFYA, ZNF514, RNF14*
**3**	GO:0045449	regulation of transcription	8.41*10^−3^	*DPF2, ZBTB22, EID2B, ZNF451, ZNF25, BANP, ZKSCAN1, ZNF514, UBN1, NR2C1, PIAS4, PRMT6, BAZ2B, TIGD7, IRAK1BP1*
	GO:0006350	transcription	1.01*10^−2^	*DPF2, ZBTB22, EID2B, ZNF451, ZNF25, BANP, ZKSCAN1, ZNF514, NR2C1, PIAS4, PRMT6, BAZ2B, IRAK1BP1*
**4**	None			
**5**	GO:0006350	transcription	2.75*10^−5^	*ERF, EGR3, ELL, NR4A2, C14ORF43, ZNF16, MEF2D, CRY2, NAB2, MNT, SERTAD3, BCL3, PER1, RARA, VGLL4, CHD6, NFIL3, SERTAD1*
	GO:0045449	regulation of transcription	3.01*10^−5^	*ERF, EGR3, ELL, NR4A2, ZNF16, C14ORF43, DLX3, MEF2D, CRY2, ID1, NAB2, MNT, SERTAD3, BCL3, PER1, RARA, VGLL4, CHD6, NFIL3, SERTAD1*
	GO:0032680	regulation of tumor necrosis factor production	3.57*10^−3^	*NOD1, BCL3, RARA*
	GO:0048511	rhythmic process	5.90*10^−3^	*EGR3, CRY2, PER1, NFIL3*
	GO:0007623	circadian rhythm	7.74*10^−3^	*EGR3, CRY2, PER1*
**6**	GO:0006916	anti-apoptosis	1.66*10^−5^	*CSF2, IER3, MCL1, HIPK3, NFKBIA, IL1B, TNFAIP3, BIRC3*
	GO:0043066	negative regulation of apoptosis	7.39*10^−5^	*CSF2, IER3, IL6, MCL1, HIPK3, NFKBIA, IL1B, TNFAIP3, BIRC3*
	GO:0043069	negative regulation of programmed cell death	8.15*10^−5^	*CSF2, IER3, IL6, MCL1, HIPK3, NFKBIA, IL1B, TNFAIP3, BIRC3*
	GO:0060548	negative regulation of cell death	8.31*10^−5^	*CSF2, IER3, IL6, MCL1, HIPK3, NFKBIA, IL1B, TNFAIP3, BIRC3*
	GO:0006915	apoptosis	1.15*10^−4^	*TRAF1, RNF144B, IER3, IL6, MCL1, HIPK3, NFKBIA, IL1B, ZC3H12A, TNFAIP3, BIRC3*

51 genes were downregulated after 31P showed an intermediate state after 1P constitute cluster 2 (Table 3). Only few significantly enriched BP were identified and their composition resembled cluster 1 with transcriptional (GO:0045449 regulation of transcription, p = 6.35×10^−4^; GO:0006350 transcription, p = 1.44×10^−4^; GO:0006355 regulation of transcription, DNA-dependent, p = 1.90×10^−2^), metabolic (GO:0051252 regulation of RNA metabolic process, p = 2.15×10^−2^), and developmental (GO:0007498 mesoderm development, p = 9.48×10^−3^) processes.

Cluster 3 (Table 3), comprising of 59 genes which were downregulated after 31P, and also showed very few enriched BP, namely GO:0045449 (regulation of transcription, p = 8.41×10^−3^), and GO:0006350 (transcription, p = 1.00×10^−2^).

Cluster 4 (Table 3) represents 37 genes which were downregulated after 1P and showed intermediate effects after 31P. No significantly enriched BP was identified.

Cluster 5 (Table 3), consisting of 59 genes upregulated after 1P only, showed BP involved in transcription (GO:0006350 transcription, p = 2.75×10^−5^; GO:0045449 regulation of transcription, p = 3.01×10^−5^), but also regulation of tumor necrosis factor production (GO:0032680, p = 3.57×10^−3^) and rhythmic processes (GO:0048511 rhythmic process, p = 5.90×10-3; GO:0007623 circadian rhythm, p = 7.73×10^−3^).

**Figure 4:**
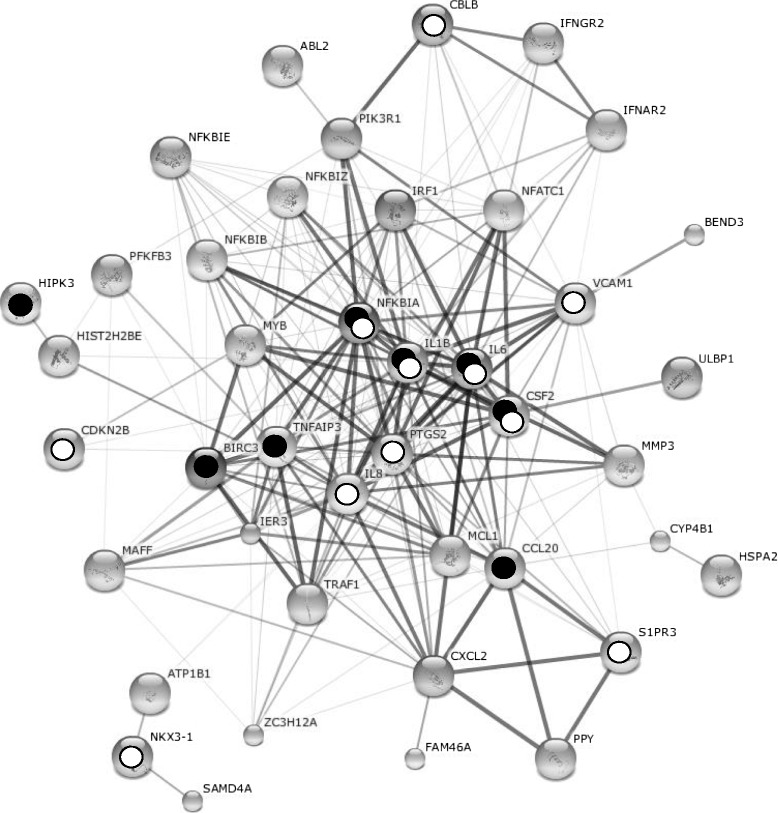
**STRING analysis of the cluster 6 from the parabolic flight experiment.** Chondrocytes were fixed after parabola 1 and 31 during a parabolic flight. In parallel, corresponding 1 *g* control samples were prepared. K-mean clustering of the resulting microarray data revealed 6 clusters of differentially expressed genes. Clusters 1–5 revealed mostly unspecific transcriptionally active genes, while cluster 6 showed a strong dominance by anti-apoptotic and cell-proliferative transcripts. Possible interactions of the corresponding proteins were visualized using the STRING software and genes involved in anti-apoptosis and cell proliferation were highlighted with white and black circles, respectively.

73 genes were upregulated only after 31P were pooled into cluster 6 (Table 3). This cluster differs considerably from the other five, as a strong emphasis of (anti)-apoptotic BP was observed. Genes such as *IL6*, *IL8*, baculoviral IAP repeat-containing protein3 (*BIRC3*), induced myeloid leukemia cell differentiation protein (*MCL1*), or TNF receptor-associated factor 1 (*TRAF1*) lead to the enrichment of BP, such as GO:0006916 (anti-apoptosis, p = 1.66×10^−5^), GO:0043066 (negative regulation of apoptosis, p = 7.39×10^−5^), GO:0043069 (negative regulation of programmed cell death, p = 8.15×10^−5^, GO:0060548 (negative regulation of cell death, p = 8.31×10^−5^), or GO:0006915 (apoptosis, p = 1.15×10^−4^) (Figure 4).

#### Quantitative real-time PCR

**Figure 5:**
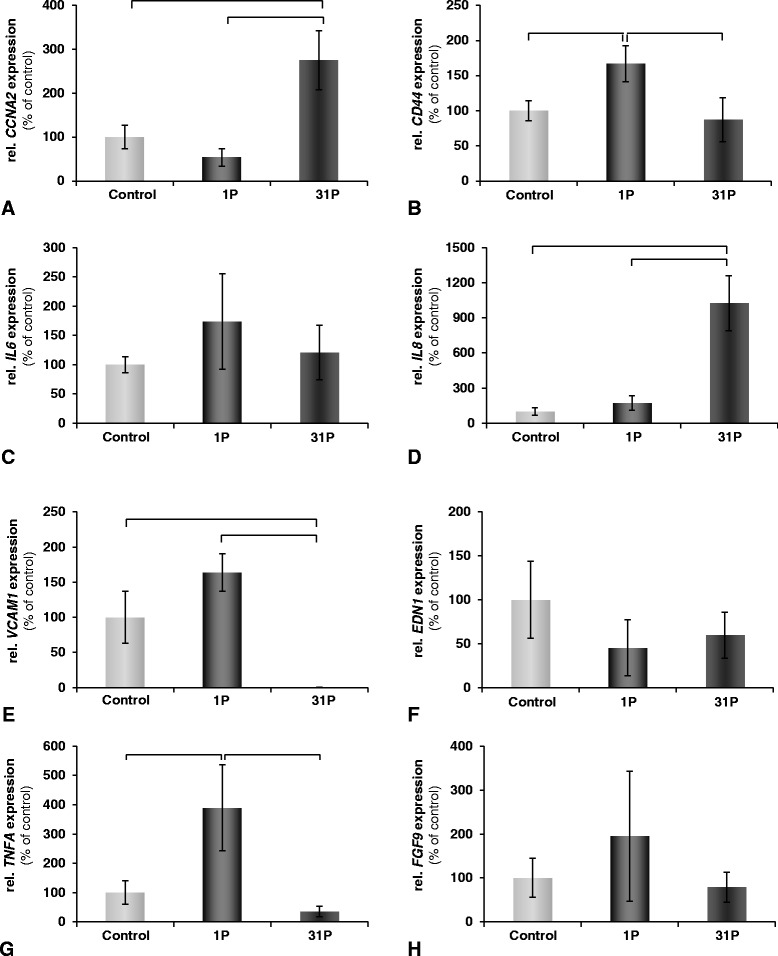
**Quantitative real-time PCR analysis of chondrocytes after exposure to parabolic flight.** Chondrocytes were taken on a parabolic flight and fixated at two different timepoints. Subsequently, qPCR analyses were performed on these samples and the corresponding 1 *g* controls. In all diagrams, the x-axis represents the experiment conditions and the y-axis the relative gene expression in % of control. Results are given as mean value ± SD. Significant changes (*p* < 0.05) are indicated by brackets above the bars. The analyzed genes were **A**: *CCNA2*; **B**: *CD44*; **C**: *IL6*; **D**: *IL8*; **E**: *VCAM1*; **F**: *EDN1*; **G**: *TNFA*; **H**: *FGF9*. 1P: fixation after first parabola; 31P: fixation after 31st parabola.

In addition to the microarray analysis, we also employed the quantitative real-time PCR technique to validate selected genes of interest. *CCNA2* as well as *IL8* were significantly upregulated only after 31P (Figure 5A, D). *CD44* and *TNFA* were significantly upregulated only after 1P (Figure 5B, G). *VCAM* showed a significant downregulation after 31P (Figure 5E). No effects were observed for *IL6*, *EDN1* and *FGF9* (Figure 5C, F, H).

## Discussion

In this study we investigated the effects of short-term real μ*g*, continuous hypergravity and vibration on human chondrocytes growing in monolayers. For these aims, we employed parabolic flight maneuvers as well as ground-based devices to expose the cells to isolated acceleration profiles and to vibrations as they occur in a combined manner during the flight conditions.

### Short-term hypergravity affects chondrocytes

The microarray analysis of chondrocytes exposed for 2 h to 1.8 *g* revealed that only a very moderate amount of genes was affected in comparison to the parabolic flight effects. It is interesting to notice, that mainly biological processes were affected, which are involved in tissue morphogenesis or skeletal system development. This is a strong indication, that hypergravity directly affects cartilage development.

It has recently been shown, that mechanical load can induce vascular endothelial growth factor A (VEGF-A) expression [21]. VEGF-A belongs to a family of growth factors comprising VEGF-A, −B, −C, −D, −E, and placenta growth factor (PGF) [22,23] and is one of the key components to control angiogenesis, the development of new vessels from existing ones. VEGF is predominantly found in osteoarthritic cartilage/chondrocytes and contributes further to the disintegration of the cartilage by inducing matrix metalloproteinases, which are able to disrupt the extracellular matrix [24,25]. We have observed a similar tendency for *VEGFA* gene expression, which was significantly elevated by a factor of 1.65, but was omitted for the GO BP analysis due to our cut-off of a Fold-Change >2. Interestingly, *MMP3* and *MMP10* transcripts were downregulated under hypergravity. We speculate that this might be due to the fact that the chondrocytes form a monolayer in the culture flasks and are not embedded in an ECM-like matrix as in their physiological environment. This strong MMP downregulation might therefore be an attempt to build up a thick ECM, and a stronger stimulus than the *VEGFA* induction.

*BMP4*, on the other hand, coding for the bone morphogenetic protein 4 (BMP-4; Additional file 1) was found to be downregulated under hypergravity. BMP-4 stimulates the synthesis of collagen type 2 and aggrecan and thus, enhaces the production of articular cartilage [26,27]. *EDN1* (endothelin 1) gene expression was observed to be enhanced (Additional file 1). It has been reported, that overexpression of endothelin 1 is associated with cartilage degeneration [28]. In contrast to this, the inhibitor of apoptosis, *BIRC3* (Additional file 1) [29], was enhanced hinting towards an improved cell survival. Furthermore, the gene expression of wingless-type MMTV integration site family, member 5A (*WNT5A*) was decreased (Additional file 1). Wnt-5a has been shown to be able to induce cartilage degradation through upregulation of MMPs [30]. All in all, it is obvious that in our setup chondrocytes are sensitive to mechanical stress by hypergravity, but at the moment, no definite answer can be given about the nature of the effect.

### Short-term vibration is detrimental to chondrocytes

In contrast to hypergravity, the effects of vibration on cultured chondrocytes were clearer. In our experimental setup, the vibrations which were transmitted into the culture flask also caused the culture medium to stir to a certain dregree, which, as we speculate, resulted in additional shear forces. Shear forces have been shown to have a negative effect on chondrocytes and cartilage [31,32]. It has been reported, that cartilage, that was treated in such a way or was degenerating, produced increased amounts of proinflammatory interleukins, such as IL-6 or IL-8 [33,34]. In our qPCR analysis we found a strong increase of both *IL6* and *IL8* (Figure 1B + D) gene expression, although no increase in IL-6 secretion (Figure 2), accompanied by decreases of *EGF, VEGFD,* and *FGF17* (Figure 1E, J, K) gene expression. The presence of these factors has been described as beneficial for cartilage development [35,36]. Taken together, our results indicate that vibration drives chondrocytes towards an inflammatory, cartilage destabilzing state.

### The influence of parabolic flight maneuvers

The microarray analysis showed, that after only 1P relatively unspecific effects on the cells were observed, mainly connected to transcription. This is an indication that the cells have perceived the change in gravity and that they were preparing their transcriptional apparatus for an altered gene expression as a reaction to this stimulus. After 31P, we observed an increase in the enrichment of anti-apoptotic genes. The qPCR analysis reflects the same tendency. Most of the investigated genes showed only transitional or no changes, such as *CD44*, *IL6*, *EDN1*, *TNFA*, and *FGF9* (Figure 5B, C, F, G, H). This seems to hint toward a short μ*g* “shock” that the cells are able to overcome very quickly. Only *CCNA2* (Figure 5A), a cyclin involved in cell cycling and proliferaton [37] and the anti-apoptotic *IL8* (Figure 5D) [38] are expressed in a manner that they exert a growth-promoting, cell-survival effect. It should be kept in mind, that these effects originate from only a short-term altered gravity (PFC) treatment and that longer exposure times have to be investigated in order to assess their significance. It is interesting to note that RPM exposure experiments resulted in increased expression of several genes responsible for cell motility, structure and integrity; control of cell growth, cell proliferation, cell differentiation and apoptosis [39] and that these results are also in very good accordance with earlier studies that also reported that chondrocytes are quite robust under μ*g* stress [40].

## Conclusions

We have shown that chondrocytes are very robust under conditions of parabolic flight maneuvers. They are able to adapt quickly to this new environment and actually profit from real μ*g* by reducing their apoptotic rate. However, they are prone to damage/injury by hypergravity and especially by vibration/shear forces. All in all, these results are very promising and are a step further along the way to understand chondrocyte growth in μ*g*, leading perhaps to new methods of scaffold-free preparation of cartilage grafts.

## Materials and methods

### Parabolic flight

The parabolic flight experiments were conducted aboard the Airbus A300 ZERO-G operated by Novespace and based in Bordeaux-Merignac, France [19,41-43]. Standard parabolic flights were performed, each with 31 parabolas in a row during the three to four hours flight. The flight manoeuver starts from the horizontal flight level followed by a 45° ascent for 20 s. During this time 1.5 *g* to 1.8 *g* are acting on the passengers and the experiments. Then the thrust is reduced and the aircraft follows the path of a parabola. The free fall (microgravity) phase starts and persists for 22 s. Afterwards, the engines are fully powered again and another phase of 1.8 *g* of 20 s terminates the parabola. Due to the aerodynamic forces and turbulences acting on the aircraft, the μ*g* quality is in the range of about 10^−2^ *g*.

### Cells and cell culture medium

Commercially available human chondrocytes (Provitro®, Berlin, Germany) were cultured in Chondrocyte Growth Medium basal (CGM, Provitro®, Berlin, Germany) supplemented with 10% fetal calf serum (Provitro®, Berlin, Germany), 100 IU penicillin/mL and 100 μ*g* streptomycin/mL (Provitro®, Berlin, Germany).

### Cell culture procedure

**Figure 6:**
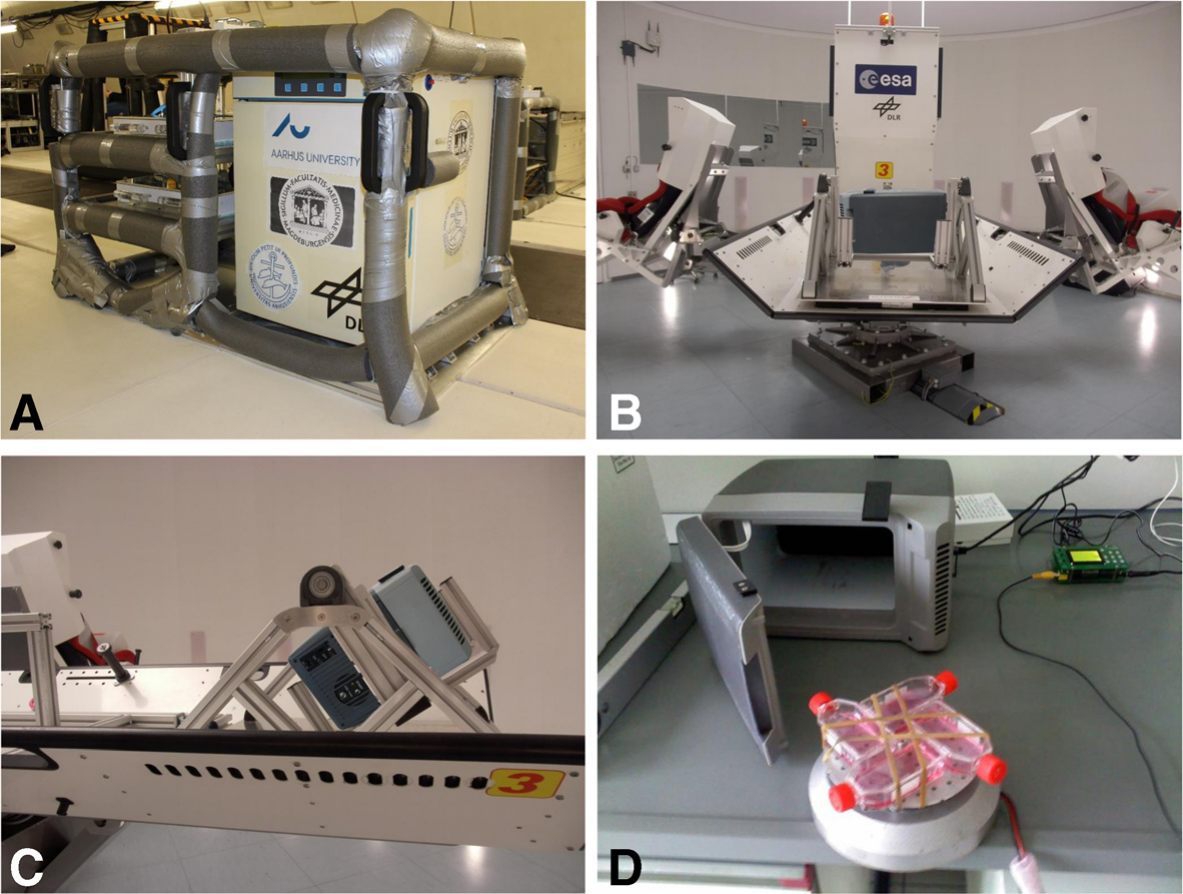
**Overview of the facilities used to expose cells to parabolic flight, hypergravity, and vibration. A**: The parabolic flight experimental rack; **B**: The Short Arm Human Centrifuge (SAHC) at the DLR, Cologne, Germany; **C**: Transportable incubator mounted on the SAHC; **D**: The Vibraplex device with T25 cell culture flasks mounted on it.

Cells were grown as published recently [19,44]. Briefly, the cells were cultured in 24 T75 cell culture flasks (75 cm^2^; Sarstedt, Nümbrecht, Germany) until subconfluent monolayers were obtained. During this time, the cells were covered by 20 mL (T-75 flasks) CGM. One half of the flasks was used as ground control cells (1 *g;* n = 12), cultured and fixed further in the laboratory, the other half was taken on the parabolic flight (n = 12). During the parabolic flight, RNA*later* (Applied Biosystems, Darmstadt, Germany) was injected via syringes containing the appropriate fixative. The syringes were connected to the T-75 flasks through a flexible tube and a 3-way-valve. One hour before each flight, the cell culture flasks were transported to the aircraft and placed into the 37°C preheated incubator on an experimental rack (Figure 6A).

### Cell fixation

The cells were fixed after the first parabola (1P) and after the 31st parabola (31P) using RNA*later* (Applied Biosystems, Darmstadt, Germany) at a ratio of 4:1 (RNA*later*:medium). After the flight, the fixative was discarded, the cells were briefly washed with PBS and covered with 10 ml of fresh RNA*later*. Subsequently, the flasks were stored at 4°C and transported to the laboratory. For the quantitative real-time PCR, we collected n = 6 T75 cell culture flasks from both parabolic flight samples (1P and 31 P) and the 1 *g* control group, the remaining flasks were used for microarray analyses.

### Hypergravity experiments

We performed experiments on the Short Arm Human Centrifuge (SAHC, DLR, Cologne, Germany) (Figure 6B), with cells cultured in T75 cell culture flasks (75 cm^2^; Sarstedt, Nümbrecht, Germany), growing in a monolayer. We installed two containers with floating mountings for the incubators on the SAHC (Figure 6C). In this configuration the T75 cell culture flasks were always exposed to a correct vertical gravity (acceleration) vector during centrifugation. By using the power supply on the SAHC the incubators were constantly heated to 37° C.

On the SAHC, we exposed the samples to a continuous hypergravity phase of 1.8 *g* of about 2 hours corresponding to the total time frame of 31 parabolas.

We designed a homogenous centrifuge profile with constant spin-up and spin-down times of each 34 seconds.

We collected n = 5 static 1 *g* controls and n = 5 1.8 *g* hyper-*g* samples for the Microarray analysis. The 1 *g* controls were cultivated in parallel in a neighboring identical incubator. Immediately after the run, the culture medium was discarded and replaced with 25 mL RNA*later* solution. For the measurement of cytokines released in the supernatant, an additional run of the SAHC was performed to obtain n = 12 static 1 *g* samples and n = 12 1.8 hyper-*g* samples for the ELISA technique.

### Vibration experiments

The detailed method was published earlier [20]. In short, the Vibraplex vibration platform (frequency range 0.2 Hz - 14 kHz) was used to create vibrations comparable to those occurring during parabolic flights (Figure 6D). Corresponding vibrations to the three phases of pull-up (1.8 *g*), free fall (μ*g*), and pull-out (1.8 *g*) were recorded and analysed by Schmidt [45]. These data were then used for the simulation experiments with the Vibraplex. For quantitative real-time PCR analyses, we collected n = 5 samples of each of the two groups (1 *g* controls and cells subjected to a vibration profile corresponding to 31 parabolas of a parabolic flight). The 1 *g* controls without vibration were grown separately in a similar incubator.

For the measurement of cytokines released in the supernatant, three additional vibration experiments were performed to obtain n = 12 static 1 *g* samples and n = 12 vibrated samples for the ELISA technique.

### RNA isolation and cDNA synthesis

After arrival in the laboratory, the RNA*later* solution on the fixed cells was replaced by PBS (Invitrogen, Darmstadt, Germany). The cells were scraped off using cell scrapers (Sarstedt, Nümbrecht, Germany), transferred to 50 ml tubes, and pelleted by centrifugation (2500 *g* for 10 min at 4°C). An RNeasy Mini Kit (Qiagen, Hilden, Germany) was used according to the manufacturer’s instructions to isolate total RNA. RNA concentrations and quality were determined spectrophotometrically at 260 nm using an Ultrospec 2100 pro Spectrophotometer (Amersham Biosciences, Freiburg, Germany). The isolated RNA had an A260/280 ratio of >1.7. cDNA designated for the quantitative real-time PCR was then obtained with the First-Strand cDNA Synthesis Kit (Fermentas, St. Leon-Rot, Germany) using 1 μg of total RNA in a 20-μL reverse transcription reaction mixture.

### Quantitative real-time PCR

Quantitative real-time PCR was used to determine the expression levels of the genes of interest. Primer Express software (Applied Biosystems) was applied to design appropriate primers with a T_m_ of ~60°C (Additional file 2). The primers were synthesized by TIB Molbiol (Berlin, Germany). All assays were run on a StepOnePlus Real-Time PCR System using the Power SYBR Green PCR Master Mix (both Applied Biosystems). The reaction volume was 25 μL including 1 μL of template cDNA and a final primer concentration of 500 nM. PCR conditions were as follows: 10 min at 95°C, 40 cycles of 30 s at 95°C and 1 min at 60°C, followed by a melting curve analysis step (temperature gradient from 60 to 95°C with +0.3°C/cycle).

If all amplicons showed one single T_m_ similar to the one predicted by Primer Express software, the PCR reactions were considered specific. Every sample was measured in triplicate, and relative quantification was effected by means of the comparative C_T_ (ΔΔC_T_) method. *18S rRNA* was used as a housekeeping gene to normalize the expression data.

### ELISA

ELISAs of IL-6, IL-8, EGF, VEGFD (R&D Systems), and FGF17 (USCN Life Science Inc.) in the cell culture supernatant from vibration and hypergravity experiments have been performed according to the protocols supplied by the manufacturer.

### Microarray analysis

Prior to the analysis, RNA integrity (RIN) was checked with the bioanalyzer. Only samples meeting the required quality were included in the analysis. The Illumina HumanWG-6_V2_0_R3 arrays have been normalized using the BeadStudio Gene Expression Module v3.3.7 and quantile normalization without background correction. After quantile normalization and exclusion of low or not expressed genes (minimum Illumina detection p-value > 0.05; performed in both analyses separately) the quality of arrays and the general expression profile has been checked by Principal Component Analysis (PCA) using Partek Genomic Suite 6.6, correlation as a dispersion matrix and normalized Eigenvector scaling. No obvious batch effect or outlier was found for the hypegravity analysis, while the outlying general expression profile of one sample from the parabolic flight experiment was removed before test statistic (for an overview: see Table 4).

**Table 4:** **Microarray analyses: projects, samples and conditions**

**Experiment**	**Conditions (Replicates)**
Hyper-g	Two Conditions: 1 g controls (N = 5) and 1.8 g (N = 4)
PFC	1 g control (N = 4), 1 parabola (N = 5), 31 parabola (N = 5 + 1 outlier)

A parametric ANOVA comparing the conditions given in Table 5 was performed. The selection criteria for the significant differential expression are also given in Table 2. Differentiation of the expression profiles was performed using K-Mean clustering. The cluster analysis was done using Partek Genomic Suite 6.3 applying the Euclidean distance function on standardized log2 signal values. K was selected according to a local minimum of the Davies Bouldin K estimation procedure. Functional aspects of the differentially expressed probes were analyzed with g: Profiler using g: SCS threshold as significance criterion and the DAVID Bioinformatics Resources 6 [46,47]. Physical and functional interactions between proteins were determined using the String platform [48] at a low confidence score of 0.15.

**Table 5:** **Microaray analyses: comparisons**

**Experiment**	**Comparisons**	**Significance criterion used**
Hyper-g	two way ANOVA(gravity vs. plate): 1 g controls and 1.8 g	5% FDR
PFC	ANOVA (parabola) 1 g control, 1 parabola, 31 parabolas	5% FDR

### Statistical analysis

All statistical analyses were performed using SPSS 16.0 software (SPSS, Inc., Chicago, IL, USA). We used either 1-way ANOVA or the Mann–Whitney U test. Differences were considered significant at the level of P < 0.05. All data are presented as means ± SD.

## Additional files

Additional file 1:
**This file provides expanded versions of Tables** 1 **and** 2 **as well as the complete overview of the GO BP analysis of the microaray data.**
Additional file 2:
**Primers used for quantitative real-time PCR.** All sequences are given in 5′-3′ direction.

## Abbreviations

BIRC3: Baculoviral IAP repeat-containing protein3
BMP: Bone morphogenetic protein
BP: Biological processes
CASP: Caspase
CAV: Caveolin
CGM: Chondrocyte growth medium
COL2A1: Collagen type 2 α 1
CTGF: Connective tissue growth factor
ECM: Extracellular matrix
EDN1: Endothelin 1
EGF: Epidermal growth factor
ELISA: Enzyme-linked immunosorbent assay
FCS: Fetal calf serum
FDR: False discovery rate
FGF: Fibroblast growth factor
FKBP1A: Peptidyl-prolyl cis-trans isomerase
FTC: Follicular thyroid cancer
GO: Gene Ontology
HAPLN1: Hyaluronan and proteoglycan link protein 1
HMGB1: High-mobility group protein B1
IL: Interleukin
ITBG1: Integrin beta 1
LAMA5: Laminin α 5
μ*g*: Microgravity
MCL1: Induced myeloid leukemia cell differentiation protein
MMP: Matrix metalloproteinase
mRNA: Messenger ribonucleic acid
NOTCH2: Neurogenic locus notch homolog protein 2
NR4A2: Nuclear receptor subfamily 4, group A, member 2
P: Parabola
PCA: Principal component analysis
PCR: Polymerase chain reaction
PGF: Placenta growth factor
PRKAA1: Protein kinase, AMP-activated, α 1
PRKCA: Protein kinase c α
PS: Parabola set
SAHC: Short arm human centrifuge
SD: Standard deviation
SOCS3: Suppressor of cytocine signaling 3
TBS: Treated parabola set
TNF: Tumor necrosis factor
TRAF1: TNF receptor-associated factor 1
RPM: Random positioning machine
TNFAIP3: TNF α-induced protein 3
TNFRSF14: TNF receptor superfamily member 14
VEGF: Vascular endothelial growth factor
VIL2: Ezrin
WNT5A: Wingless-type MMTV integration site family, member 5A.
