# Supplementary material for: Differential gene expression of human chondrocytes cultured under short-term altered gravity conditions during parabolic flight maneuvers
Source: Cell Commun Signal. 2015 Mar 20;13:18. doi: 10.1186/s12964-015-0095-9 (PMC4369370; doi:10.1186/s12964-015-0095-9)
Supplement: Additional file 2: — Primers used for quantitative real-time PCR. All sequences are given in 5′-3′ direction. [file 12964_2015_95_MOESM2_ESM.docx]

**Additional file 2: Primers used for quantitative real-time PCR**

| ***Gene*** | ***Primer Name*** | ***Sequence*** |
| --- | --- | --- |
| *18S rRNA* | 18S-F | GGAGCCTGCGGCTTAATTT |
|  | 18S-R | CAACTAAGAACGGCCATGCA |
| *CAV2* | CAV2-F | GATCCCCACCGGCTCAAC |
|  | CAV2-R | CACCGGCTCTGCGATCA |
| *CCNA2* | CCNA2-F | GCAGCAGAGGCCGAAGAC |
|  | CCNA2-R | CCAAGGAGGAACGGTGACA |
| *CD44* | CD44-F | ACCCTCCCCTCATTCACCAT |
|  | CD44-R | GTTGTACTACTAGGAGTTGCCTGGATT |
| *CTGF* | CTGF-F | ACAAGGGCCTCTTCTGTGACTT |
|  | CTGF-R | GGTACACCGTACCACCGAAGAT |
| *EDN1* | EDN1-F | ACTTCTGCCACCTGGACATCA |
|  | EDN1-R | CTCCAAGGCTCTCTTGGACCTA |
| *EGF* | EGF-F | TGCCAGCTGCACAAATACAGA |
|  | EGF-R | TCTTACGGAATAGTGGTGGTCATC |
| *FGF9* | FGF9-F | TATCCAGGGAACCAGGAAAGAC |
|  | FGF9-R | CTGACCAGGCCCACTGCTATAC |
| *FGF17* | FGF17-F | CCGAGGACGGCAACAAGT |
|  | FGF17-R | ACTTCTCACTCTCAGCCCCTTTG |
| *IL6* | IL6-F | CGGGAACGAAAGAGAAGCTCTA |
|  | IL6-R | GAGCAGCCCCAGGGAGAA |
| *IL8* | IL8-F | TGGCAGCCTTCCTGATTTCT |
|  | IL8-R | GGGTGGAAAGGTTTGGAGTATG |
| *IL15* | IL15-F | CATCCAGTGCTACTTGTGTTTACTTCT |
|  | IL15-R | CCAGTTGGCTTCTGTTTTAGGAA |
| *PRKAA* | PRKAA-F | AATCCTTCTGTGATTAGCCTTTTG |
|  | PRKAA-R | GATCCTCAGGAAAGAGATATTTTGGAA |
| *PRKCA* | PRKCA-F | TGGGTCACTGCTCTATGGACTTATC |
|  | PRKCA-R | CGCCCCCTCTTCTCAGTGT |
| *TNFA* | TNFA-F | TGATCGGTCCCAACAAGGA |
|  | TNFA-R | TGGGCTACGGGCTTGTCA |
| *VCAM1* | VCAM1-F | CATGGAATTCGAACCCAAACA |
|  | VCAM1-R | GGCTGACCAAGACGGTTGTATC |
| *VEGFA* | VEGFA-F | GCGCTGATAGACATCCATGAAC |
|  | VEGFA-R | CTACCTCCACCATGCCAAGTG |
| *VEGFD* | VEGFD-F | TGCAGGAGGAAAATCCACTTG |
|  | VEGFD-R | CTCGCAACGATCTTCGTCAA |
| *VIL2* | VIL2-F | GAAGTGCACAAGTCTGGGTACCT |
|  | VIL2-R | CTCCCACTGGTCCCTGGTAAG |

All sequences are given in 5’-3’ direction
